# Medical Items

**Published:** 1916-07

**Authors:** 


### MEDICAL ITEMS

### FOR THE PROTECTION OF THE PATIENT.

In this day of sophistication and substitution the earnest them to have—and nothing else. Especially is this so in regard to remedies of exceptional quality, such as Glay's Glycerine onic Comp. Numerous imitations of this reliable tonic are constantly being foisted on the unsuspecting, and when as a consequence of the patient failing to get the original "Gray's," the expected results do not materialize, the doctor's skill and ability are apt to be questioned. For the protection of his patient and in justice to himself, the physician should invariably write for "Gray's" as follows:

R. Gray's Glycerine Comp.

(Purdue Frederick Co.) One bottle—16 oz.

By thus specifying an original package, the painstaking physician will safeguard his patients and insure his results.

---

### THE LIVER IN AUTOTOXIC ILLS.

The liver, as the largest gland in the body and the one that is called upon to do the most work, is to a certain extent both the "clearing house" and the "depository" of the body's nutritional reserve. It is easy to understand, therefore, how even a slight disturbance of its functions, may be followed by serious consequences throughout the whole organism.

Realizing this it is little wonder that the trained clinician is so keen and prompt to take steps to prevent the continuation of hepatic derangements. Undoubtedly it is zeal in this direction that has led so many physicians to prize Chionia, for they have found it a remedy that can be relied upon not only to restore and maintain hepatic activity, but happily without exciting excessive or objectionable bowel movement. The exceptional therapeutic efficiency of Chionia therefore, in all functional disorders of the liver has made it one of the most valuable and practically useful remedies at the command of the practitioner who realizes the paramount importance of assuring hepatic activity, especially in ills of an auto-toxic character.

### RESTORING THE PHYSIOLOGIC ACTIVITY OF THE BOWELS

There are scores of drugs listed in the materia medicas and pharmacopoeias which have some direct or indirect action on the bowels. They exert their influence in various ways—mechanically, physiologically and medicinally—and all have more or less merit—as their use and recommendation go to indicate.

It should be remembered, however, that catharsis and purgation are properly never anything but emergency measures—to be used only "on the spur of the moment" when quick eliminative action is needed; as a consequence of which cathartic and purgative drugs have no place in the routine or systematic treatment of constipation. Gentle stimulation of the bowels, on the other hand, by means of mild but effective laxatives offers the more rational means of treating bowel inactivity, the commonest of the human ills, and should always be called upon when permanent benefits are sought. The effects of such measures will be prompt and decided, without pain, griping or distress of any kind, and since they are brought about by proper stimulation of physiological processes, they are naturally more prolonged and persistent.

Remedies that can accomplish such results are few and far between. Prunoids is one of the best of them and its gentle but thoroughly satisfactory action in all forms of constipation, stasis and even intestinal atony, through its influence not only on peristalsis, but also on secretory activity have made it the remedy of choice for this class of cases by thousands of physicians in all parts of the world. An opportunity to test its value will be given to all physicians who address the Sultan Drug Co., St. Louis, Mo.

---

"I used the samples of Tongaline Liquid and Tongaline and Lithia Tablets for my wife, who was suffering from a severe attack of the grippe, with such success that she made a prompt and thorough recovery."

### AN ALTERATIVE OF LONG SERVICE.

It is mainly in chronic skin and glandular diseases that alteratives have found their most distinct field of usefulness, for these are conditions aggravated and continued by impaired nutrition and elimination, in the correction of which alteratives show what potent remedial forces they are. Among the alteratives IODIA (Battle) has long enjoyed professional favor and in this will be found a striking demonstration of its value, for no class of drugs are put to a more rigid test than alteratives, so its long continued use by physicians is the best evidence that it meets the demands made upon it. IODIA (Battle) will show its power in chronic skin diseases, glandular involvements and in other states indicating the corrective influence of an alterative agent. A distinct advantage offered by IODIA is that it may be continued over long periods without causing distress.

---

### TREATMENT OF APOPLEXY

In the December issue of Surgery, Gynecology and Obstetrics, Dr. Charles M. Remsen of Atlanta has a monograph upon the early radical treatment of Apoplexy. He collates the experience of several investigators as to the locations of apoplexies and he outlines the diagnostic signs that conform with the autopsies and operative findings.

Armed with this information he advocates the early relief from cerebral pressure, and drainage of the blood before damage can be increased either by the pressure or the changes that occur in the clots themselves. He reports a case greatly benefitted even after the sixth day and urges that surgery be resorted to while there is still a chance to make it beneficial.

---

"On account of the extraordinary action of Tongaline on the liver, the bowels, the kidneys and the pores, I have found it a really wonderful medicine, because it expels so rapidly and thoroughly the poisons and germs which are the cause of rheumatism, grippe, lumbago etc."

